# Novel circular RNA circSLIT2 facilitates the aerobic glycolysis of pancreatic ductal adenocarcinoma via miR-510-5p/c-Myc/LDHA axis

**DOI:** 10.1038/s41419-021-03918-y

**Published:** 2021-06-24

**Authors:** Hua Guan, Wei Luo, Yuping Liu, Mingfei Li

**Affiliations:** 1 Department of Health Management, Sichuan Provincial People’s Hospital, University of Electronic Science and Technology of China, Chengdu, Sichuan China; 2 Department of Stomatology, Sichuan Provincial People’s Hospital, University of Electronic Science and Technology of China, Chengdu, Sichuan China; 3 Department of Hepatobiliary Surgery, Sichuan Provincial People’s Hospital, University of Electronic Science and Technology of China, Chengdu, Sichuan China

**Keywords:** Cancer metabolism, Cancer metabolism

## Abstract

Increasing evidence has indicated the great diagnostic and therapeutic potentials of circular RNAs (circRNAs) in human cancers. Although the biological roles of circRNAs in pancreatic ductal adenocarcinoma (PDAC) have been partially annotated, the potential regulatory mechanism of circRNAs in PDAC tumorigenesis remains poorly understood. Here, our study found that the novel circRNA circSLIT2 was significantly upregulated in PDAC tissues and cells. Clinically, ectopic high-expression of circSLIT2 was correlated with unfavorable prognosis of PDAC patients. Functional experiments demonstrated that circSLIT2 promoted the aerobic glycolysis and proliferation of PDAC cells in vitro, and circSLIT2 knockdown inhibited tumor growth in vivo. Mechanistically, circSLIT2 acted as miRNA sponge to target miR-510-5p/c-Myc axis. Furthermore, c-Myc bound with the promoter region of lactate dehydrogenase A (LDHA) to activate the transcription. Collectively, present findings reveal that circSLIT2/miR-510-5p/c-Myc/LDHA axis participates in the aerobic glycolysis and carcinogenesis of PDAC, and may act as a promising therapeutic target.

## Introduction

Pancreatic ductal adenocarcinoma (PDAC) is one of the most serious malignancies with extraordinarily high fatality rate and terrible prognosis [1, 2]. Although great advances have made in PDAC treatment, the 5-year survival rate is fabulously less than 6% [3]. As in many human tumors, the variation in genetic and epigenetic of oncogenes and tumor suppressor genes could modulate the PDAC tumorigenesis [4]. Therefore, it is urgent to identify an effective method and novel therapy targets for PDAC.

Circular RNAs (circRNAs) are a group of primarily non-coding RNAs, which are generated by backsplicing events without 5′ and 3′ terminal structures [5, 6]. The biogenesis or origination of circRNAs is mediated by the back-splicing. As compared with linear mRNAs, circRNAs are covalently bonded through 5′–3′ polarity or poly(A) tail and resistant to the exonucleases [7, 8]. CircRNAs participate in the regulation of PDAC tumorigenesis. For instance, circRNA_0007334 is significantly upregulated in PDAC and serves as competing endogenous RNA to relive the MMP7 through sponging miR-144-3p/577 [9]. A novel circ_0030235 is upregulated in PDAC tissues compared with matched normal tissue specimens, and the overexpression is closely correlated to higher tumor stage and positive lymph node invasion [10]. Certainly, the deepgoing molecular mechanisms of circRNA in the PDAC are still elusive.

Aerobic glycolysis, also known as Warburg effect, is typically acknowledged as a major hallmark of human cancer [11–13]. The distinct energy metabolism manner is an essential issue during tumorigenesis and provides rapid energy and fundamental substance source, promoting the uncontrolled proliferation and invasion of PDAC cells [14, 15]. Therefore, the energy metabolism disorder significantly participates in the tumorigenesis of PDAC, which may serve as a potential therapeutic target.

Up to now, circRNAs exert critical roles in the PDAC pathophysiological processes, however, how the Warburg effect is directly regulated by circRNA is largely unknown. In the present research, we identified a novel circRNA circSLIT2 derived from SLIT2 exon4–exon2 (hsa_circ_0009113, 216 bp). Functionally, circSLIT2 was significantly upregulated in PDAC tissue and cells. High expression of circSLIT2 was correlated with an unfavorable prognosis for PDAC patients. Collectively, these findings reveal that circSLIT2/miR-510-5p/c-Myc/LDHA axis participates in the aerobic glycolysis and carcinogenesis of PDAC, and may act as a promising therapeutic target.

## Results

### CircRNA circSLIT2 was identified to be upregulated in PDAC

**Fig. 1:**
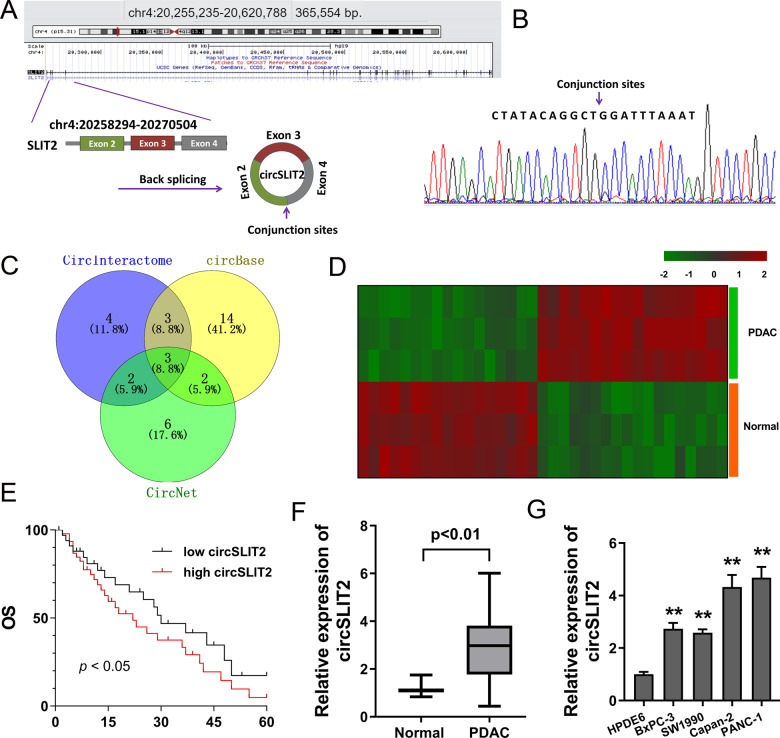
CircRNA circSLIT2 was upregulated in PDAC tissue and cells. **A** A novel circRNA circSLIT2 (hsa_circ_0009113) was generated from the exon 4 to exon 2 with 216 spliced length. **B** Sanger sequencing demonstrated the full length of circSLIT2 and the conjunction sites of exon 4 and exon 2. **C** Venn diagram showed the candidate circRNAs from the circBase (http://www.circbase.org/), CircInteractome (https://circinteractome.nia.nih.gov/) and CircNet (http://syslab5.nchu.edu.tw/CircNet/). **D** The dysregulated circRNA expression profiles in the PDAC tissue and normal tissue were analyzed using GEO dataset (GSE69362). **E** Kaplan–Meier survival curves and log-rank test were performed to evaluate the survival of PDAC individuals with high or low circSLIT2 expression. **F** RT-qPCR illustrated the circSLIT2 expression in the PDAC specimens and normal specimens. **G** RT-qPCR illustrated the circSLIT2 expression in PDAC cell lines.

Originally, our study utilized the GEO dataset [16] to discover the dysregulated circRNAs profiles and then performed RT-qPCR to identify these candidate circRNAs. Then, as the verification experiment went on, several circRNAs were excluded and we finally chose the novel circSLIT2 (hsa_circ_0009113). CircSLIT2 was generated from exon 4 to exon 2 with 216 spliced length (Fig. 1A). Sanger sequencing demonstrated the full length of circSLIT2 and the conjunction sites of exon 4 and exon 2 (Fig. 1B). Venn diagram showed the candidate circRNAs from the circBase (http://www.circbase.org/), CircInteractome (https://circinteractome.nia.nih.gov/), and CircNet (http://syslab5.nchu.edu.tw/CircNet/) (Fig. 1C). Besides, the dysregulated circRNA expression profiles in the PDAC tissue and normal tissue were analyzed using GEO dataset (GSE69362), showing the potentially dysregulated circRNAs and confirmed previous findings (Fig. 1D). Kaplan–Meier survival curves and log-rank test were performed to evaluate the survival of PDAC individuals, showing the poor prognosis of high circSLIT2 PDAC groups as compared to the low group (Fig. 1E). In the PDAC specimens and normal specimens (Table 1), RT-qPCR illustrated that circSLIT2 was remarkedly upregulated in the PDAC tissue specimens (Fig. 1F). In PDAC cell lines, RT-qPCR illustrated that circSLIT2 was significantly overexpressed as compared to the normal cells (Fig. 1G). Collectively, circRNA circSLIT2 was identified to be upregulated in PDAC tissue and cells and indicated the poor prognosis of PDAC patients.

### CircSLIT2 boosted the aerobic glycolysis of PDAC cells

**Fig. 2:**
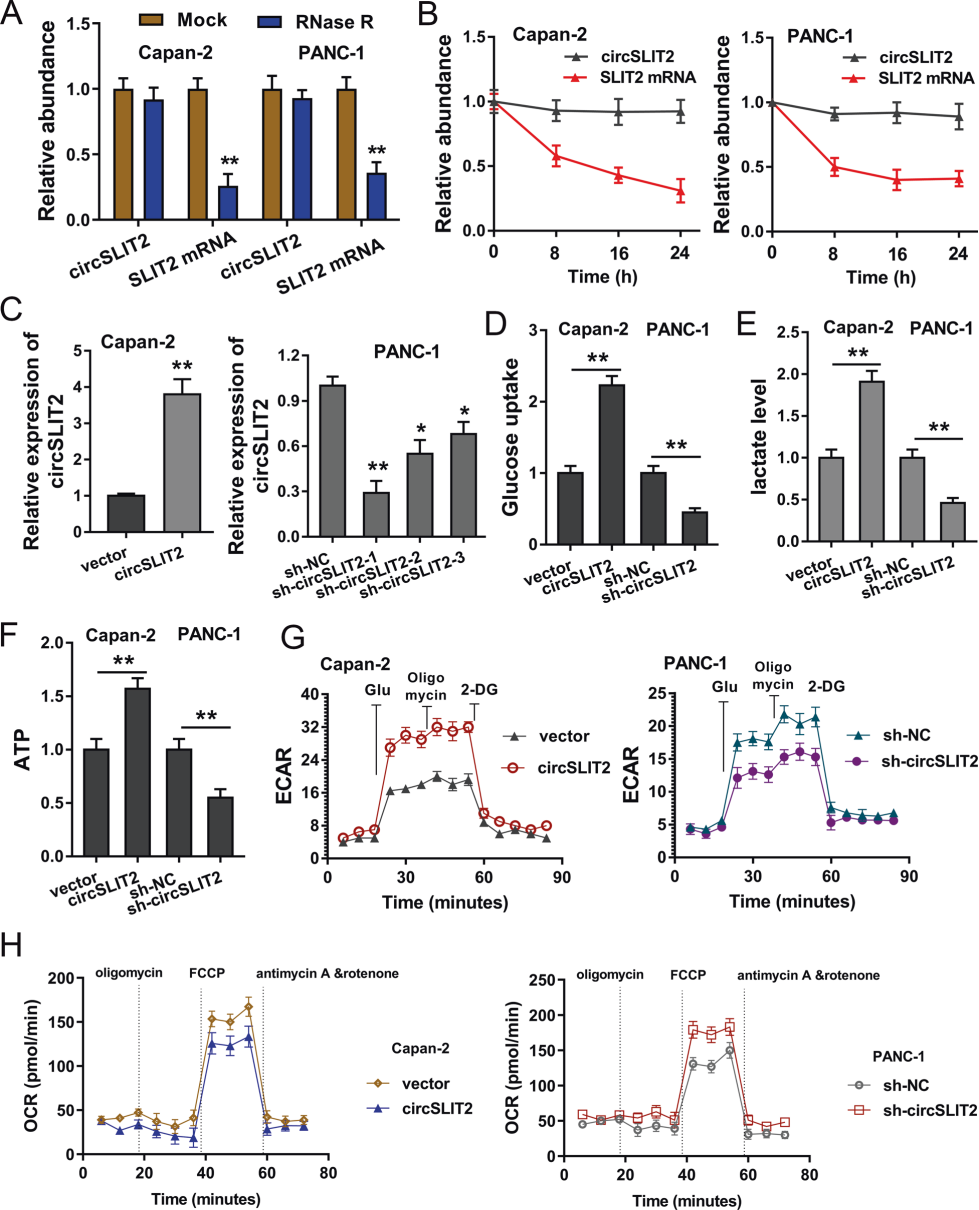
CircSLIT2 boosted the aerobic glycolysis of PDAC cells. **A** RT-qPCR showed the expression of circular form transcripts circSLIT2 and linear form transcripts in PDAC cell treated with RNase R. **B** RT-qPCR indicated the circular form transcripts circSLIT2 and linear form transcripts SLIT2 mRNA in PDAC cells administrated with actinomycin D. **C** The overexpression and silencing of circSLIT2 transfection were constructed using the plasmids or oligonucleotides transfection in Capan-2 and PANC-1 cells. **D** The abundance of glucose uptake was measured. **E** The production quantity measured by lactate analysis in Capan-2 and PANC-1 cells. **F** ATP analysis indicated the ATP output in PDAC cells. **G** Extracellular acidification rate (ECAR) assay and (**H**) oxygen consumption rate (OCR) assay demonstrated the glycolytic capacity of PDAC cells. **p < 0.01; *p < 0.05.

In the cellular functional experiments, we found that the circular form transcripts circSLIT2 survived greater than the linear form transcripts when treated with RNase R (Fig. 2A). RT-qPCR indicated that circular form transcripts circSLIT2 level was much more stable than the linear form transcripts (SLIT2 mRNA) when the PDAC cells were administrated with actinomycin D (Fig. 2B). The overexpression and silencing of circSLIT2 transfection were constructed using the plasmids or oligonucleotides transfection (Fig. 2C). The abundance of glucose uptake was measured and showed that circSLIT2 promoted the glucose absorption quantity in PDAC cells (Fig. 2D). The production quantity measured by lactate analysis found that circSLIT2 promoted the lactate production level in Capan-2 and PANC-1 cells (Fig. 2E). ATP analysis indicated that circSLIT2 accelerated the ATP output in PDAC cells (Fig. 2F). Extracellular acidification rate (ECAR) assay and oxygen consumption rate (OCR) assay demonstrated that circSLIT2 accelerated the glycolytic capacity of PDAC cells (Figs. 2G, H). Thus, in the cellular analysis, we concluded that CircSLIT2 boosted the aerobic glycolysis of PDAC cells.

### CircSLIT2 regulated the proliferation and tumor growth of PDAC cells

**Fig. 3:**
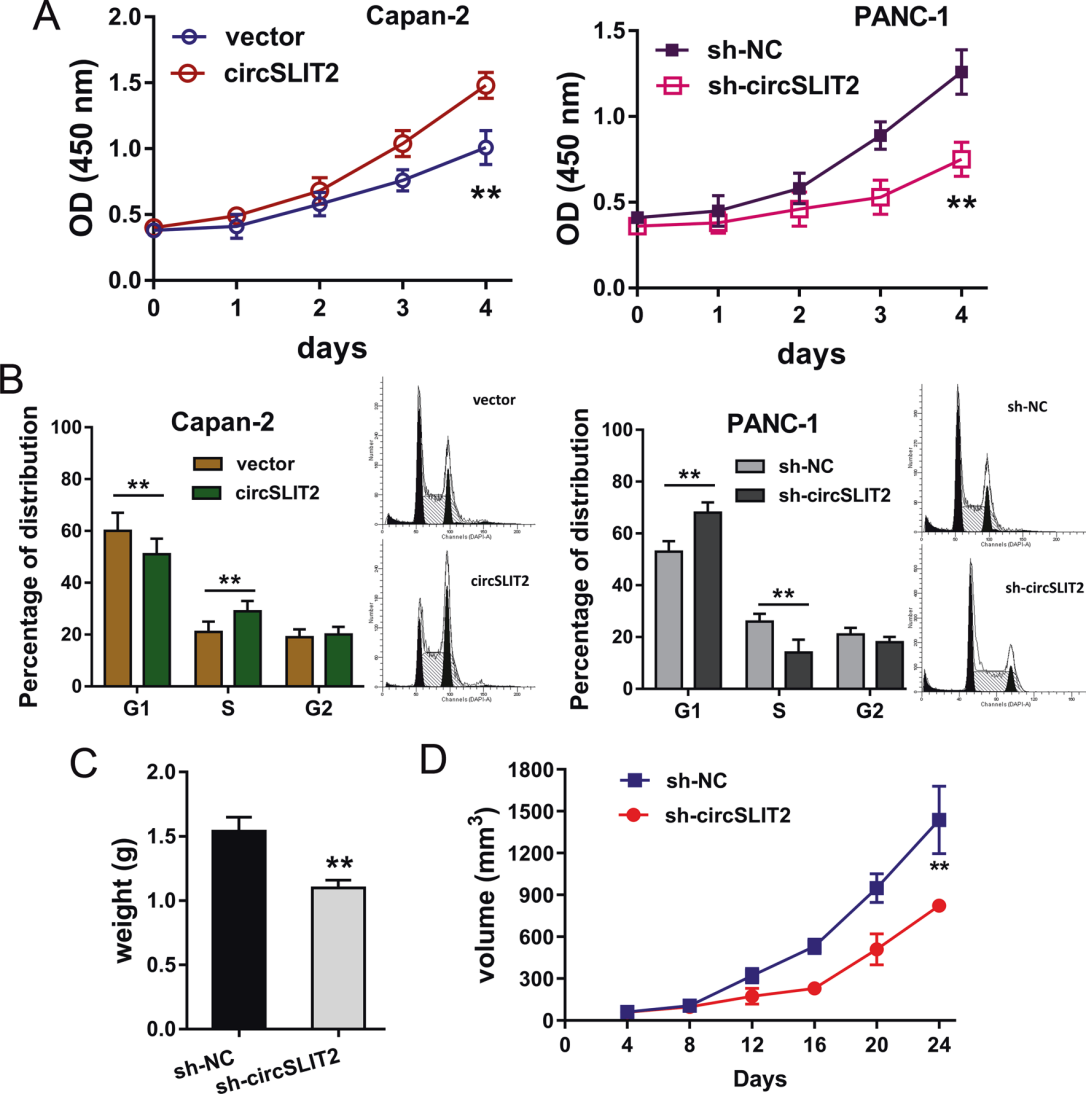
CircSLIT2 regulated the proliferation and tumor growth of PDAC cells. **A** CCK-8 assay indicated the proliferation of Capan-2 cells transfected with circSLIT2 overexpression and PANC-1 cells transfected with circSLIT2 knockdown. **B** Flow cytometric for the cycle analysis demonstrated the cycle progression or cycle arrest at G0/G1 phase in Capan-2 or PANC-1 cells. In vivo mice xenograft assay showed that circSLIT2 knockdown in PANC-1 cells inhibited the tumor growth weight (**C**) and volume (**D**) of PANC-1 cells. **p < 0.01.

Subsequently, more cellular experiments were performed to identify the biological roles of circSLIT2 for PDAC cells. CCK-8 assay indicated that circSLIT2 overexpression promoted the proliferation of Capan-2 cells, and circSLIT2 knockdown repressed the proliferation of PANC-1 cells (Fig. 3A). Flow cytometric for the cycle analysis demonstrated that circSLIT2 overexpression accelerated the cycle progression of Capan-2 cells, and circSLIT2 knockdown induced the cycle arrest at G0/G1 phase (Fig. 3B). In vivo mice xenograft assay showed that circSLIT2 knockdown inhibited the tumor growth, including weight and volume, of PANC-1 cells (Fig. 3C, D). Thus, in the cellular analysis, we concluded that circSLIT2 regulated the proliferation and tumor growth of PDAC cells.

### MiR-510-5p served as the target of circSLIT2 in PDAC cells

**Fig. 4:**
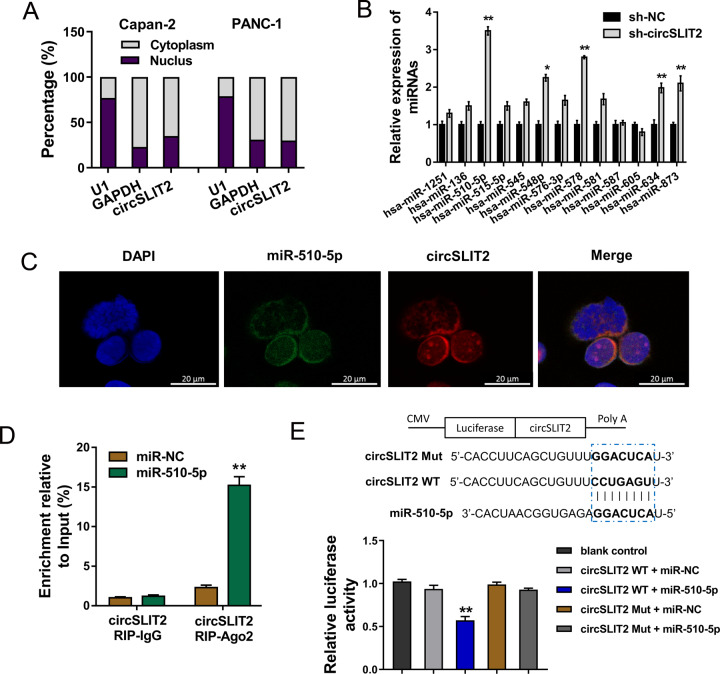
MiR-510-5p served as the target of circSLIT2 in PDAC cells. **A** Subcellular fraction analysis indicated the cytoplasmic or nuclear distribution of circSLIT2. **B** Expression of miRNAs predicted by bioinformatics analysis (https://circinteractome.nia.nih.gov/) using RT-qPCR in PANC-1 cells transfected with circSLIT2 knockdown. **C** RNA fluorescence in situ hybridization (RNA-FISH) unveiled the distribution of miR-510-5p (labeled by FAM probe) and circSLIT2 (labeled by Cy3 probe). **D** RNA immunoprecipitation (RIP) assay was performed in PANC-1 cells to confirm the association between circSLIT2 and miR-510-5p. **E** The wild type (WT) correspond with miR-510-5p and mutant (Mut) vector for circSLIT2 were constructed. Luciferase reporter assay indicated the covalent binding within miR-510-5p and circSLIT2 wild type (WT) or mutant (Mut) vector. *p < 0.05; **p < 0.01.

Subcellular fraction analysis indicated that circSLIT2 was mainly located in the cytoplasmic distribution (Fig. 4A). For the mechanism investigation, we detected the expression of miRNAs predicted by bioinformatics analysis using RT-qPCR and these results unveiled that multiple miRNAs were dysregulated in PANC-1 cells transfected with circSLIT2 knockdown (Fig. 4B). We found that miR-510-5p presented a significantly correlation with sh-circSLIT2. Importantly, we performed further functional assay to identify the roles of these candidate miRNAs. Moreover, RNA fluorescence in situ hybridization (RNA-FISH) unveiled that miR-510-5p (labeled by FAM probe) was mainly located in the cytoplasmic distribution, and circSLIT2 (labeled by Cy3 probe) was similarly located in the cytoplasmic distribution (Fig. 4C). The association of circSLIT2 and miR-510-5p was confirmed using RNA immunoprecipitation (RIP). Results demonstrated that miR-510-5p overexpression group promoted the enrichment level of circSLIT2 in Ago2 RIP group comparing to IgG RIP group (Fig. 4D). Among these candidate miRNAs, miR-510-5p was one of the most dysregulated one. Moreover, the wild type (WT) corresponding to miR-510-5p and mutant (Mut) vector for circSLIT2 were constructed. Luciferase reporter assay indicated that miR-510-5p closely interacted with the circSLIT2 wild type through covalent binding (Fig. 4E). Collectively, the evidence concluded that miR-510-5p served as the target of circSLIT2 in PDAC cells. Actually, other miRNAs and other targets of miR-510-5p worth exploring in the future.

### c-Myc served as the target of miR-510-5p and promoted LDHA transcription

**Fig. 5:**
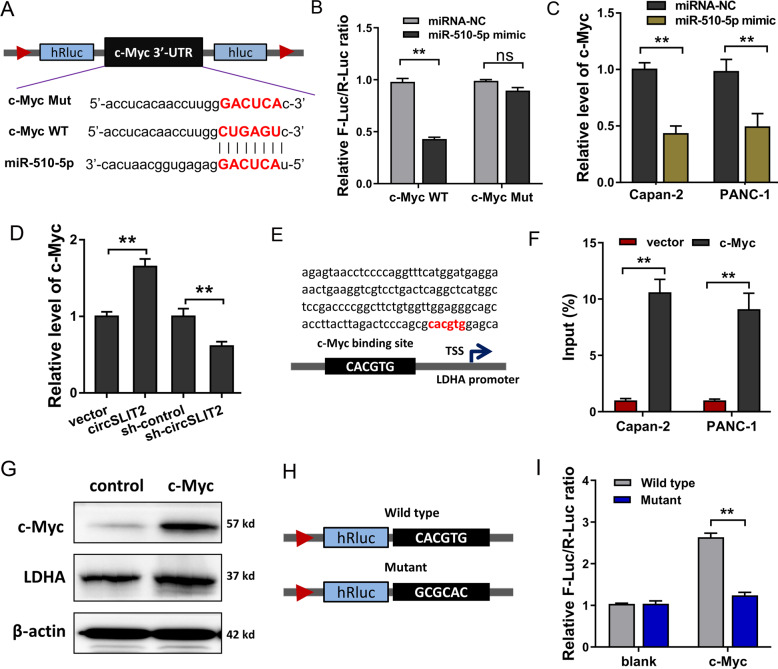
c-Myc served as a direct target of miR-510-5p and promoted LDHA transcription. **A** miR-510-5p harbored the covalent complementary binding sites with c-Myc mRNA at 3′-UTR. **B** Luciferase reporter analysis unveiled the combination within miR-510-5p mimics and c-Myc mRNA wild type (WT) or mutant (Mut) sequences at 3′-UTR. **C** RT-qPCR showed the expression of c-Myc mRNA level in Capan-2 and PANC-1 cells administrated with miR-510-5p mimics transfection. **D** RT-qPCR showed the expression of c-Myc mRNA level in Capan-2 and PANC-1 cells administrated with circSLIT2 overexpression. **E** Schematic diagram illustrated the binding sites of transcription factor c-Myc at the promoter region of LDHA near transcriptional start site (TSS). **F** Chromatin immunoprecipitation (ChIP) elucidated the enrichment of LDHA promoter binding sites sequence in the precipitated complex with c-Myc antibody. **G** Western blot analysis uncovered the LDHA protein level after c-Myc overexpression transfection. **H** Luciferase reporter vectors for LDHA promoter binding sites, including wild type (WT) and mutant (Mut) sequences, were synthesized. **I** Luciferase reporter analysis elucidated the interaction of c-Myc and LDHA promoter region. **p < 0.01.

Furthermore, we investigated whether a protein served as the target of miR-510-5p. After filtration, we identified a potential target for miR-510-5p. miR-510-5p harbored the covalent complementary binding sites with c-Myc mRNA at 3′-UTR (Fig. 5A). Luciferase reporter analysis unveiled that miR-510-5p mimics closely combined with the wild type sequences of c-Myc mRNA 3′-UTR (Fig. 5B). In Capan-2 and PANC-1 cells, miR-510-5p mimics transfection significantly repressed the expression of c-Myc mRNA level (Fig. 5C). Besides, circSLIT2 overexpression could upregulate c-Myc mRNA level, and circSLIT2 knockdown repressed it (Fig. 5D). In the promoter region of LDHA, transcription factor c-Myc harbored the direct binding sites near the transcriptional start site (TSS) (Fig. 5E). Chromatin immunoprecipitation (ChIP) elucidated that the predetermined sites sequences were remarkedly precipitated by c-Myc antibody (Fig. 5F). Western blot analysis uncovered that c-Myc overexpression augmented the LDHA protein level (Fig. 5G). Luciferase reporter vectors for LDHA promoter binding sites, including wild type and mutant sequence, were synthesized (Fig. 5H). Luciferase reporter analysis elucidated that c-Myc closely interacted with LDHA promoter region, indicating the transcription potential of c-Myc for LDHA (Fig. 5I). Collectively, these findings supported that c-Myc served as the target of miR-510-5p and promoted LDHA transcription.

## Discussion

Recently, increasing quality of circRNAs have been discovered and identified in multiple tumor tissues and cell lines with the help of next-generation sequencing technology (NGS) [17, 18]. Due to characteristics of tissue-specificity and cell-specificity, circRNAs have diverse distribution and biological roles. Besides, due to the characteristic of covalent closed loop, circRNAs could competitively survive in cellular microenvironment as comparing to linear transcripts [19]. It has been reported that circRNAs play critical roles in multiple malignant behaviors including proliferation, migration, metastasis, and chemoresistance [20, 21]. CircRNAs could not only exert regulatory functions in various biological processes, but also act as promising diagnostic markers in human cancers.

In this study, our team demonstrated a novel circRNA in PDAC. CircRNA circSLIT2 is derived from SLIT2 exon4-exon2 (hsa_circ_0009113, 216 bp). Functionally, circSLIT2 was significantly upregulated in PDAC tissue and cells. CircSLIT2 high-expression indicated the unfavorable prognosis for PDAC patients. For the cellular biological function experiments, we found that circSLIT2 promoted the Warburg effect (aerobic glycolysis), including glucose uptake, lactate production ad ATP accumulation. Besides, circRNA circSLIT2 promoted the proliferation and regulated the cycle progression in vitro and the tumor growth in vivo. Overall, these results elucidate that circRNA circSLIT2 regulated the Warburg effect and tumorigenesis in PDAC cells.

To further explore the underlying molecular mechanism by which circSLIT2 regulated the downstream effector targets in PDAC cells. Currently published literature reports that a fraction of circRNA could coordinate with their host gene, however, some don’t. Because of the cytoplasmic location, circSLIT2 might serve as a sponge for miRNA as most other circRNAs. Given that the functions of circRNAs are dependent on their subcellular localization, we assume that circSLIT2 may perform as a miRNA sponge. To screen the potential target of miRNAs, we identified the candidate miRNAs predicted by CircInteractome (https://circinteractome.nia.nih.gov/). CircSLIT2 could harbor the miR-510-5p to alleviate its abundance in PDAC cells. Moreover, miR-510-5p targeted the 3′-UTR of c-Myc mRNA to negatively regulate its expression. So far, the evidence illustrated that circSLIT2/miR-510-5p/c-Myc axis functioned as a critical regulator for the PDAC Warburg effect and tumorigenesis.

**Fig. 6:**
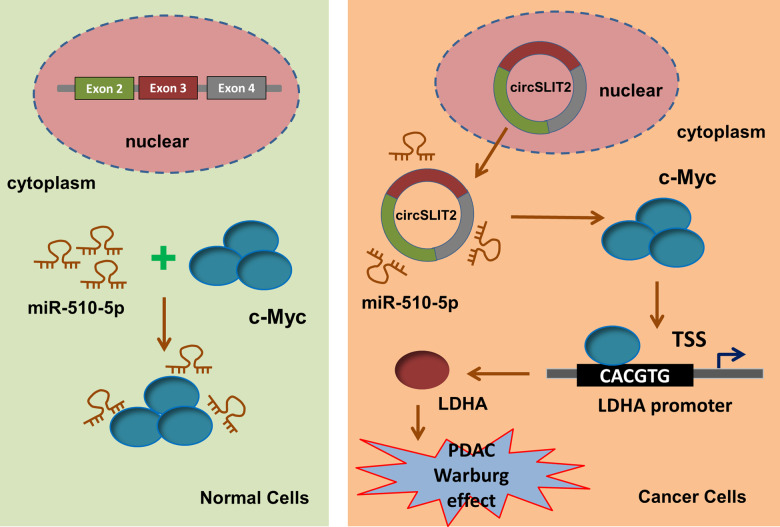
circSLIT2/miR-510-5p/c-Myc/LDHA axis promoted the tumorigenesis and Warburg effect in PDAC cells.

Warburg effect, also known as aerobic glycolysis, is typically acknowledged as a major hallmark of human cancer [22]. The aerobic glycolysis could provide rapid energy and material supplement for tumor growth. In human tumorigenesis, glycolysis plays an important role and this pathological feature has been identified to be regulated by circRNAs. For example, circFOXP1 (hsa_circ_0008234) is significantly upregulated in gallbladder cancer tissues and positively associated with poor prognosis in patients. CircFOXP1 interacts with PTBP1, which binds to the 3′-UTR region and coding region of PKLR mRNA (UCUU sequences) to impair the decay of PKLR mRNA, thereby promoting Warburg effect in gallbladder cancer progression [23]. Exosome-delivered circRNA ciRS-122 (hsa_circ_0005963) promotes glycolysis to reduce drug susceptibility in chemosensitive cells in colorectal cancer through sponging PKM2-targeted miR-122 [24]. In present research, we identified the critical regulation by which circSLIT2/miR-510-5p/c-Myc/LDHA axis promotes the tumorigenesis and Warburg effect in PDAC cells (Fig. 6).

Trustily, there are several weaknesses in this research due to experimental condition limitations and schematic design insufficiency. For instance, whether the circSLIT2/miR-510-5p/c-Myc/LDHA pathway takes effect in tissue in vivo, including xenograft tumor tissues, still needs further verification. Moreover, the correlation between circSLIT2 expression and downstream target molecules (miR-510-5p, c-Myc, LDHA) in the human clinical samples might also provide valuable theoretical support for this finding. Furthermore, circSLIT2 functions as a miRNA sponge in the tumorigenesis and Warburg effect through sponging miR-510-5p. In addition to miR-510-5p/c-Myc/LDHA pathway, there could be other miRNA-protein axis in the progression.

**Table 1:** Relationship between circSLIT2 and PDAC patients’ clinicopathological characteristic.

Characteristic		circSLIT2		*p*
		*L* = 14	*H* = 16	
Gender				
Male	17	8	9	0.623
Female	13	6	7	
Age				
<60	16	8	8	0.751
≥60	14	6	8	
Differentiation				
Well	6	2	4	0.437
Moderate	16	7	9	
Poor	8	5	3	
TNM				
I–II	12	8	4	0.013*
III–IV	18	6	12	
Lymph node metastasis				
No	11	7	4	0.008*
Yes	19	7	12	

*L* Low; *H* High.

*P < 0.05.

Here, our findings describe a regulation in which circSLIT2 functions as a competitive endogenous RNA to sponge miR-510-5p, thereby relieving the expression of c-Myc and, ultimately leading to the upregulation of in LDHA. Collectively, this study reveals that circSLIT2/miR-510-5p/c-Myc/LDHA axis significantly participates in the Warburg effect and carcinogenesis of PDAC, and may act as a promising therapeutic target.

## Materials and methods

### Tissue samples

Two groups of tissue samples, including PDAC tissue (thirty samples) and matched adjacent normal tissue, were recruited from patients who underwent surgical resection. These tissues were immediately stored in liquid nitrogen after surgery in Sichuan Provincial People’s Hospital. All of the tissues in the study were diagnosed by pathologist. This study had been approved by the Ethics Committee of Sichuan Provincial People’s Hospital. All patients had signed the consent form.

### Cell culture

Normal human pancreatic ductal epithelial cell line (HPDE6) and PDAC cells (PANC-1, Capan-2, SW1990, BxPC-3) were provided from American Type Culture Collection (ATCC, Manassas, VA, USA). Cells were cultured in Dulbecco’s modified Eagle’s medium (Gibco BRL, Grand Island, NY, USA) containing 10% fetal bovine serum (FBS, HyClone, Invitrogen) in humidified incubator at 37 °C in 5% CO_2_.

### Plasmid construction and transfection

To obtain the circSLIT2 overexpression, the front-back circular frames were especially synthesized to construct the circular transcripts. Overexpression plasmids for circSLIT2 were constructed using pCD5-ciR vector (Greenseed Biotech, Guangzhou, China). Specific shRNAs targeting circSLIT2 (sh-circSLIT2) and their control shRNAs (sh-NC) were designed and synthesized by Genepharm (Suzhou, China). The stably transfected cells were detected to test the transfection efficiency. MiRNAs mimic and their control was designed and synthesized by RiboBio (Guangzhou, China). Transfections were conducted by using Lipofectamine 2000 (Invitrogen, NY, USA). The sequences are listed in Table S1. The transfection efficiency of overexpression and knockdown was detected using RT-qPCR.

### Quantitative Real-Time PCR

Total RNA sample (1000 ng) was reversely transcribed to cDNA using High Capacity cDNA Reverse Transcription Kit (Applied Biosystems, Darmstadt, Germany). Amplification and qPCR process were performed using SYBR Green PCR kit (TaKaRa, Dalian, China) on Applied Biosystems 7300 Fast real-time PCR system (Applied Biosystems, Waltham, MA, USA). β-actin was used as endogenous control. The sequences of primers used in PCR are listed in Table S1.

### CircRNA microarray analysis

CircRNAs expression analysis performed and the dysregulated circRNA expression profiles in the PDAC tissue and normal tissue according to Gene Expression Omnibus (GEO) dataset (GSE69362). The data set consisted of three paired primary PDAC tissues and surrounding normal tissues were included. The raw GSM files of GSE69362 were analyzed using R software.

### RNase R and actinomycin D treatment

Total RNA (2 μg) was extracted from PDAC cells. The extracted RNA was administrated with RNase R (3 U/μg, Epicenter Technologies, Madison, WI, USA). For RNA stability assay, extracted RNA from PDAC cells was incubated with Actinomycin D (Act-D, Catalog #A9415, Sigma-Aldrich, St. Louis, MO, USA) at 5 μg/ml for the indicated time. The RNA expression levels of SLIT2 mRNA and circSLIT2 were detected by RT-qPCR.

### Glucose uptake, lactate production, ATP levels analysis, extracellular acidification rate (ECAR) and oxygen consumption rate (OCR)

For glucose uptake, colorimetric glucose assay kit (BioVision, Milpitas, CA, USA) was performed to detect glucose concentration according to the manufacturer’s instructions. For lactate production, lactate assay kit (K627, BioVision) was performed to detect the lactate concentration in the PDAC cells lysis according to the manufacturer’s instructions. For ATP levels, ATP assay kit (S0026, Beyotime) was used to detect the intracellular ATP levels in cellular extraction. For ECAR, glucose (10 mM), oligomycin (1 μM), and 2-deoxyglucose (50 mM) were administrated in each well sequentially at indicated time point. For OCR analysis, oligomycin, mitochondrial uncoupler carbonyl cyanide p-trifluoromethoxy phenylhydrazone (FCCP), and antimycin A and rotenone (Rote/AA) were administrated in each well sequentially.

### CCK-8 proliferation and flow cytoplasm apoptosis

Proliferation of PDAC cells was measured by the CCK-8 assay kit (Dojindo Japan). Briefly, cells were seeded in 96-well culture plates. 10 μl of CCK-8 reagent was added for incubation and the absorbance was measured at 450 nm. For apoptosis, cells were washed and then resuspended in Binding Buffer (100 μl) and stained 5 μl of FITC Annexin V and 5 μl of propidium iodide (PI) at room temperature in the dark. The apoptosis was analyzed using apoptosis Kit (KeyGen, Nanjing, China) and subjected to flow cytometric analysis.

### Western blot analysis

The total protein of PDAC cells was exacted with RIPA buffer and separated by 10% SDS-PAGE, then transferred to PVDF membrane (Bio-Rad, CA, USA). PVDF membranes were blocked with 5% non-fat milk powder and incubated with primary antibodies against c-Myc (Abcam, ab185656) and LDHA (Abcam, ab101562) at 4 °C overnight and GAPDH (1:5000) (Cell Signaling Technology) at room temperature for 2 h. Band blots were quantified by densitometry (Quantity One software; Bio-Rad) and ImageJ software.

### Subcellular analysis and RNA fluorescence in situ hybridization (RNA-FISH)

The nuclear/cytosolic fraction was separated using PARIS kit (Life Technologies, Carlsbad, CA, USA) in PDAC cells according to the manufacturer’s instructions. For RNA-FISH assay, Cy3-labeled circSLIT2 probe and FAM-labeled miR-510-5p probe were synthesized by GenePharma (Shanghai, China). The in situ hybridization was carried out using the circRNA Hybridization Kit (Foco, Guangzhou, China) according to the manufacturer’s instructions. Fluorescence detection was performed with a confocal laser-scanning microscope (Leica, Wetzlar, Germany).

### Dual-luciferase reporter assay

The wild type sequences of circSLIT2 and c-Myc 3′-UTR and their corresponding mutant sequences targeting miR-510-5p binding sites were synthesized. These sequences were subcloned into luciferase reporter vector psiCHECK2 (Promega, Madison, WI, USA) respectively. Vectors were co-transfected into 293 T cells by Lipofectamie (2000) (Invitrogen) according to the manufacturer’s protocol. The relative luciferase activities were calculated by Dual Luciferase Assay Kit (Promega, Madison, WI, USA) in accordance with the manufacturer’s protocols.

### RNA immunoprecipitation (RIP)

RIP assay was performed using the Magna RNA immunoprecipitation kit (Millipore, MA, USA) according to the manufacturer’s instructions. PANC-1 cells were transfected with miR-510-5p or miR-NC were lysed by lysis buffer and then incubated with immunoprecipitation buffer containing Ago2 antibody-coated magnetic beads. IgG and Input acted as controls. After elution, the extracted circRNA and miRNA expression was determined by qRT-PCR.

### Chromatin immunoprecipitation (ChIP) assay

ChIP assays were performed using the SimpleChIP® Enzymatic Chromatin IP Kit (Cell Signaling Technology, Inc., Danvers, MA, USA). PDAC cells were subjected to shear the cross-link DNA into fragments in 4% formaldehyde at room temperature for 15 min. After sonication, chromatin fragments were immunoprecipitated with c-Myc-specific antibody (Abcam, ab185656) at 4 °C overnight. Normal rabbit IgG antibody (Santa Cruz Biotechnology, CA, USA) functions as control. Immunoprecipitation was collected and the DNA was then analyzed by real-time PCR.

### Animal studies

Animal studies were approved by the Institute Animal Care and Use Committee of Sichuan Provincial People’s Hospital according to institutional guidelines and approved protocols. Male BALB/c nude mice (5 weeks old) were purchased from Slac Laboratory Animal Center (Shanghai, China). For xenograft transplantation, 1 × 10^6^ cells in 100 μl suspension in containing 50% Matrigel were subcutaneously injected into the flank of mice. The tumor length and width were respectively measured and the volume was calculated using the formula = 0.5 × long and short diameter × short diameter^2^. Tumor weight was measured after neoplasm excision.

### Statistical analysis

Data are expressed as mean ± SD. Statistical analysis was performed using GraphPad (San Diego, CA, USA). Student’s *t*-test (two-tailed) was used for two-group comparisons and one-way analysis of variance (ANOVA) was used for multiple group comparisons. *P* value less 0.05 was considered statistically significant (*p < 0.05, **p < 0.01).

## Supplementary information

Supplement Table S1
