# Supplementary material for: Novel circular RNA circSLIT2 facilitates the aerobic glycolysis of pancreatic ductal adenocarcinoma via miR-510-5p/c-Myc/LDHA axis
Source: Cell Death Dis. 2021 Jun 24;12(7):645. doi: 10.1038/s41419-021-03918-y (PMC8225611; doi:10.1038/s41419-021-03918-y)
Supplement: Supplementary file 1 — Supplement Table S1 [file 41419_2021_3918_MOESM1_ESM.docx]

**Table S1**. Sequences of shRNA and qRT-PCR primers.

|  | 5’-3’ |
| --- | --- |
| circSLIT2 | forward, 5’-ACTGCGTTTAAACAGAAATCACCT-3'  reverse, 5'-GACCAGCAAAATCTGTCTTCGT-3’ |
| sh-SLIT2-1 | 5’-ATGGGTTCCACACCTGGAAGATTA-3’ |
| sh-SLIT2-2 | 5’-CCCAGATCATGGTTTTGGCACCTT-3’ |
| sh-SLIT2-3 | 5’-TTGGGCCAACTCTAAATGGGAAAGT-3’ |
| miR-510-5p | forward, 5’-GCATAATGGTTCAGCATGTG-3’  reverse, 5’-GCATCATGGCAGCATTTACA-3’ |
| c-Myc | forward, 5’-CAGGACTGTATGTGGAGCGGCTT-3'  reverse, 5'-GCGAGCTGCTGTCGTTGAGAGGG-3’ |
| LDHA | forward, 5’-ATGGCAACTCTAAAGGATCAGC -3’  reverse, 5’-CCAACCCCAACAACTGTAATCT-3’ |
| β-actin | forward, 5’-AAGCCACCCCACTTCTCTCTAA-3’  reverse, 5’-AATGCTATCACCT CCCCTGTGT-3’ |
